# A Revised Surgical Strategy for the Distal Tibiofibular Interosseous Osteochondroma

**DOI:** 10.1155/2020/6371456

**Published:** 2020-05-07

**Authors:** Huarui Yang, Kangquan Shou, Shijun Wei, Zhi Fang, Qiwen Hu, Qiong Wan, Yi Yang, Tongzhu Bao

**Affiliations:** ^1^Department of Orthopedic, The First College of Clinical Medical Science, China Three Gorges University & Yichang Central People's Hospital, Yichang 443002, China; ^2^Department of Orthopaedics, General Hospital of Central Theater Command, Wuhan, China; ^3^Department of Gynaecology and Obstetrics, The First College of Clinical Medical Science, China Three Gorges University & Yichang Central People's Hospital, Yichang 443002, China

## Abstract

Osteochondroma is one of the most common benign bone tumor; however, the surgical treatment still remains a challenge for those that occur at the distal tibiofibular interosseous location. Previously, the transfibular approach has been successfully described, but the potential damage of the syndesmosis would give rise to the instability of the ankle joint and thus may result in the unfavorable long-term outcome. Here, a revised strategy which can protect the syndesmotic complex is introduced. From 2010 to 2017, eleven patients with the distal tibiofibular interosseous osteochondroma who underwent the revised surgery were collected. The distal fibular osteotomy and posterior tibial osteotomy were performed to keep the inferior syndesmosis intact for better stability of the ankle joint. Both the anterior tibiofibular ligaments (AITFL) and posterior tibiofibular ligaments (PITFL) have been preserved successfully, and thus, the stability of the ankle joint has been maintained due to our strategy. The VAS and AOFAS scores were utilized to assess the clinical outcome and function. Postoperatively, all the patients were pain-free and were able to wear the appropriate shoes at the last follow-up. Preoperative and postoperative AOFAS scores were 93.63 ± 6.91 and 47.27 ± 5.27 (*P* < 0.05), respectively. Moreover, the average VAS score was 1.73 ± 0.27 (compared with preoperative as 7.45 ± 2.15, *P* < 0.05), demonstrating obvious improvement after the operation. To our best knowledge, this is the first time to perform the resection of the distal tibial interosseous osteochondroma involving the fibula without interrupting the inferior syndesmotic complex especially the AITFL and PITFL. We believe that this strategy may pave a new way for optimized clinical outcome for these patients with distal tibiofibular interosseous osteochondroma. This clinical trial study is registered with number ChiCTR1900024690.

## 1. Introduction

Osteochondroma is defined as a benign lesion with a bony structure covered by a cartilaginous cap [1]. It is reported that it accounts for 40% of all benign bone tumors [2]. Most of the osteochondromas are asymptomatic and usually discovered accidentally by routine physical examination. Osteochondromas localized around the foot and ankle are thought to be difficult to manage, despite their benign nature. Notably, the osteochondroma involving of both of the distal tibia and fibula is particularly uncommon, maybe accounting for less than 1% of 1937 excised lesions reported in one series [3]. Basing on the its particular location, this rare type of osteochondromas may give rise to significant symptoms accompanied with several complications such as the bony deformation of tibia and fibula or varus/valgus deformities of ankle joint, blocking of joint motion, syndesmotic problems, and neurologic or vascular compromise [4]. To this end, it has been acknowledged that excision of the tumor is recommended for those who are symptomatic or have potential damage in the surrounding tissues to prevent the potential pathological fracture or ongoing ankle deformity and to treat the symptoms as well [5].

So far, the clinical outcome after the surgery is not favorable both for the surgeon and patients. Notably, the poor outcome may be attributed to its disruption for the ankle joint, especially, the inferior tibiofibular syndesmosis [3]. In other words, inappropriate surgical intervention may give rise to the damage for syndesmotic complex, resulting in the instability of the ankle joint. Thus, how to perform the surgical procedure to resect the tumor mass thoroughly without disturbing or interrupting the syndesmosis complex is worth to be introduced and assessed.

Normally, the anterior tibiofibular inferior ligament (AITFL) is the weakest one of the four syndesmotic ligaments and is the first to yield to forces that create an external rotation of the fibula around its longitudinal axis [6]. Thus, it is pretty important to protect this crucial construct during the surgery; otherwise, the ligament reconstruction needs to be taken into account to restore the anatomic ankle joint biomechanics, which would be of particular interest in younger patients or high-demand athletes [7, 8].

In this study, for the first time, we introduced a revised strategy to remove the osteochondroma without disturbing the inferior syndesmosis by using distal fibular osteotomy and posterior tibial osteotomy to keep the syndesmostic complex, especially AITFL and PITFL, intact during the surgical procedure. We evaluated the clinical functional outcome of the patients and proved that this new surgical technique could accomplish the thorough resection of tumor as well as preserve the inferior tibiofibular syndesmosis complex, thus significantly improving the stability of the ankle joint for the better long-term function of the ankle joint and clinical prognosis.

## 2. Methods

### 2.1. Patients

From October 2010 to January 2017, a total of 11 patients with distal tibiofibular interosseous osteochondroma who were admitted to the China Three Gorges University & Yichang Central People's Hospital were collected. There were 7 male and 4 female patients with an average of 27.52 ± 11.36 years (range, 18–43 years), and all the demographic and clinical data including age, gender, the affected side, symptoms were recorded (Table 1). The inclusion criteria were as follows: (1) consistent with the diagnosis of interosseous osteochondroma located at distal tibiofibular area; (2) all the patients were treated with surgery; and (3) postoperative pathological results were confirmed as osteochondroma. The exclusion criteria consisted of as follows: (1) patients with severe systemic diseases who cannot undergo surgery (such as shock, damage to vital organs, poorly controlled diabetes mellitus, serious cardiovascular and cerebrovascular diseases; (2) poor blood coagulation profile; and (3) those patients who refused to be followed-up. The definition and the pathological finding of the osteochondroma were confirmed according to the previous reports [3].

All the patients performed X-ray with anteroposterior (AP view) and lateral position preoperatively, including a mortise view as well. Radiographs were evaluated by an experienced orthopaedic surgeon to determine the radiographic finding. In addition, a computed tomography (CT) plain scan with three-dimensional reconstruction and magnetic resonance imaging (MRI) examination were also acquired if necessary to assess the involvement of the lesion. All the procedures were approved by the Ethics Committee of our hospital, and all the patients signed a written consent before the surgery.

### 2.2. Operative Procedure

**Figure 1:**
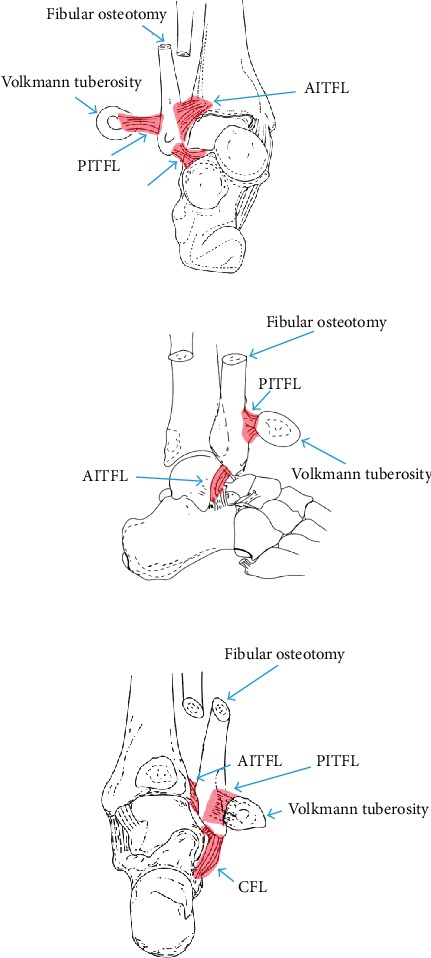
Illustration of the detailed surgical procedure. AITFL: anterior tibiofibular ligament; PITFL: posterior tibiofibular ligament; CFL: calcaneofibular ligament; ATFL: anterior talofibular ligament.

After the lateral approach was performed, the peroneus muscle was retracted away to expose the distal fibula. The locking plate was placed and every hole of screw was prepared prior to the distal fibular osteotomy to ensure the later anatomical reduction of the fibular fragment when the lesion resection was achieved. Then the osteotomy of distal fibula was performed right above the level of the inferior syndesmosis or according to the location of tumor mass, preventing the damage brought to the syndesmosis complex. Next, the distal fragment of fibula was rotated anteromedially to expose the tumor mass. If the tumor mass encroached the lateral side of tibia, the posterior osteotomy of the distal tibia (osteotomy of Volkmann's tuberosity) was performed to expose the intact margin of the lump, and the Kirschner wires were used to fix the retracted Volkmann tuberosity back; thus, the inferior tibiofibular syndesmosis complex especially the AITFL was successfully preserved. After the tumor mass was removed thoroughly, the fragment of fibula was placed back to its original location to achieve the anatomical reduction. If necessary, the cannulated screw (diastasis screw) was utilized to stabilize the inferior tibiofibular joint. Autologous iliac bony grafting would be taken into account when the remaining cave was too large to prevent the nonunion or malunion. Further histology was performed to confirm the diagnosis of osteochondroma with no malignant transformation (Figure 1).

### 2.3. Postoperative Management

Postoperatively, the patient was mobilized with plaster for nonweight bearing for 4-6 weeks. Next, the patients were encouraged to commence active exercises with a full range of motion at the ankle joint. Meanwhile, gradual transition from partial to full weight bearing was performed under proper protection. At final routine clinical follow-up, the functional outcome and the range of motion were recorded.

### 2.4. Functional Assessment

The scoring system of the American Orthopaedic Foot and Ankle Society (AOFAS) (excellent, 90–100; good, 75–89; fair, 50–74; and poor, <50) [9] and Visual Analogue Scale (VAS) (good, 0-3; fair, 4-6; and poor, 7-10) [10] were used to evaluate the functional outcome.

### 2.5. Statistical Analysis

All values were presented as mean ± standard deviation (SD) and analyzed by repeated measures (ANOVA). A *P* value < 0.05 was regarded as statistically significant.

## 3. Results

In this study, all 11 patients were successfully followed-up with average of 1.9 ± 0.4 years. All patients were shown to resume with full activities at the last follow-up, and no evidence of recurrence was observed. Moreover, all the patients were pain-free and were able to wear the appropriate shoes such as casual shoes or sneakers; however, they complained no proper shoes were available for them before the operation.

All the lesion in this study was further confirmed as osteochondroma, which have been previously proved to be benign [3]. All the distal anterior tibiofibular ligaments have been preserved successfully, and thus, the stability of the ankle joint has been maintained due to our revised strategy. Most of the cases showed the encroachment of the osteochondroma into the distal tibia and fibula with the apparent depression of the cortex, whereas several cases indicated that the talus was also involved (data not shown).

At 3 months postoperative follow-up, the average AOFAS score was 80.99 ± 4.33 (compared with preoperative as 47.27 ± 5.27, *P* < 0.05). The good rate was 54.5% with 6 cases rated as good, 4 as fair, and only 1 as poor (Tables 2 and 3). With regard to the VAS score, there were 7 cases claimed as good, 3 cases as fair, and 1 case as poor, respectively. Specifically, the average VAS score was 3.73 ± 0.33 compared with the preoperative measurement as 7.45 ± 2.15, demonstrating obvious improvement after the operation (*P* < 0.05, Tables 2 and 3).

Furthermore, we assessed the long-term outcome at 1 year after operation and the significant improvement was observed from the comparison between 3 months and 1 year postoperative follow-up (80.99 ± 4.33 vs. 93.63 ± 6.91 for AOFAS, *P* < 0.05; 3.73 ± 0.33 vs. 1.73 ± 0.27 for VAS score, *P* < 0.05), indicating the favorable outcome with long-term observation (Tables 2 and 3).

**Figure 2:**
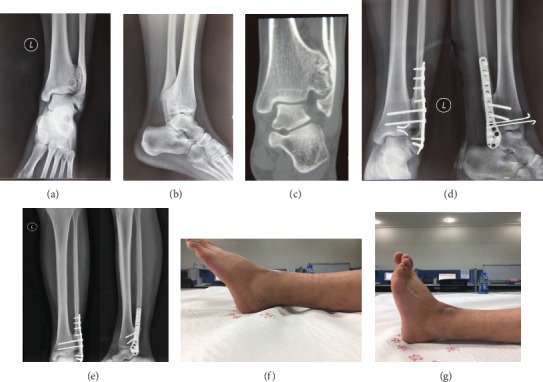
Preoperative ((a) anteroposterior; (b) lateral; (c) CT scan with one layer) and postoperative ((d) day 1 after surgery; (e) 6 weeks after surgery, the Kirschner wires have been removed) radiographs from a 32-year-old man. It is noted that the posterior tibial osteotomy was used to retract the Volkmann tuberosity, which was reduced back anatomically to preserve the inferior tibiofibular syndesmosis intact. The lateral photograph at the final follow-up showed good range of motion of ankle joint (FnnG).

**Figure 3:**
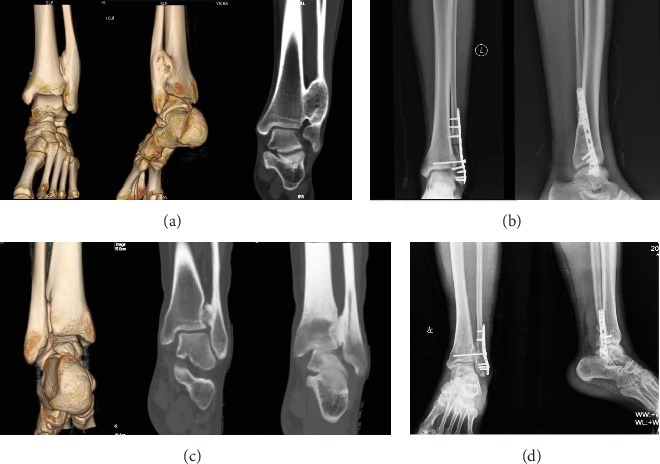
Preoperative ((a) CT scan with three-dimensional reconstruction and one layer) and postoperative ((b) anteroposterior and lateral X-ray) radiographs from a 29-year-old woman. Preoperative ((c) CT scan) and postoperative ((d) anteroposterior and lateral X-ray) radiographs from a 24-year-old man.

Complications included a 9.09% (1/11) infection with superficial tissue and 9.09% (1/11) nonunion rate (one patient with nonunion located at distal fibular osteotomy). The infections were solved with enhanced wound dressing accompanied with the application of antibiotics. No deep tissue infection or osteomyelitis was observed. The fibular nonunion patient was managed with revision surgery by replacing the internal fixation coupled with autologous bone graft, and the ultimate union was observed at 6 months after the revision surgery. The typical cases are shown in Figures 2 and 3.

## 4. Discussion

The osteochondromas discussed here were referred as those located at either the metaphysis or the junction of the metaphysis of the tibia and fibula. Although these has been explored by several studies for the last decades [5, 11], few reports have addressed the optimal strategy to protect the syndesmotic complex especially the distal anterior tibiofibular ligament when the resection was performed. In this study, we utilized a revised technique to preserve this crucial construct by the simple method of distal tibial osteotomy. We proved that the new surgical technique is able to accomplish the thorough resection of the tumor and preserve the syndesmosis complex, thus significantly improving the stability of the ankle joint and the long-term functional prognosis. This is a significant beneficial finding, given that the most postoperative complication such as pain in the patients with this particular disease was proved to be derived from the instability of the ankle joint [12].

It has been reported that osteochondromas are considered as the most common benign bone tumors [2]. They are thought to be derived from the periosteum with small cartilaginous nodules. Even osteochondromas are asymptomatic when they are discovered occasionally, progressive enlargement may still give rise to side effect including skeletal deformity, nerve compression, pathological fracture, etc. Therefore, it has been acknowledged that excision of the tumor is recommended for those who are symptomatic or have potential damage in the surrounding tissues. The aim of the surgical treatment is to prevent the pathological fracture, correct ankle deformity, eliminate the possibility of malignant transformation, and improve the symptoms [5]. However, increasing concerns have emerged considering that resection of the lesion may lead to the disruption of the equilibrium that have been already established between the lesion and the ankle, giving rise to the unbalanced mechanics and instability of the ankle joint. More importantly, the inferior syndesmosis plays an important role in stability for the ankle joint. Thus, simple resection of the tumor mass without reconstruction of the syndesmosis is obsolete. Therefore, it is worthy to evaluate the modified surgical intervention for the perseveration of the syndesmostic complex during the tumor resection.

In our study, we introduced a revised strategy to keep the syndesmostic complex intact during the surgical procedure by distal fibular osteotomy and distal anterolateral tibial osteotomy to preserve the distal anterior tibiofibular ligament (AITFL). Normally, the AITFL is the weakest one of the four syndesmotic ligaments and is the first to yield to forces that create an external rotation of the fibula around its longitudinal axis [6]. Thus, it is pretty important to protect this crucial construct during the surgery; otherwise, the ligament reconstruction needs to be taken into account to restore the anatomic ankle joint biomechanics, which would be of particular interest in younger patients or high-demand athletes [7, 8]. Briefly, we used the osteotomy technique to tract the “Tillaux-chaput” fragment anterolaterally to perform the complete resection of tumor mass possible and then inserted the fragment back with the fixation by two Kirschner's wires. With the help of this procedure, we realized the preservation of the syndesmotic complex especially the AITFL, probably relating to the favorable clinical outcome after the operation. Compared with the previous reports, our findings demonstrated the better clinical outcome and more favorable function for the patients. Previously, Gopa et al.[11] reported the distal tibial interosseous osteochondroma involving the fibula and presented a transfibular excision of mass and reconstruction of distal fibula using a square nail. However, the tumor did not involve the inferior syndesmosis, thus making the resection without considering the reconstruction of the syndesmotic complex possible. Similarly, several other studies described the transfibular excision and focused on the complete bone healing of the fibula after the osteotomy instead of the preservation of the inferior syndesmotic complex [2]. Another study reported the arthrodesis of distal tibiofibular joint to resolve the instability of the ankle joint after the tumor resection [5], but this procedure may give rise to a stiff ankle joint and reduced gait/function for the patients, which would be not acceptable for those patients with high demand of sports activity.

In a word, our findings proved that the new strategy by using the distal tibial osteotomy was able to achieve the preservation of the inferior tibiofibular syndesmosis especially the AITFL, leading to the better functional outcome and favorable prognosis for the patients.

## 5. Conclusion

Herein, we introduced a new strategy to maintain the distal anterior tibiofibular ligament intact, thus protecting the integrity of the syndesmosis complex, which is believed to be the keystone of the stability of the ankle joint. With the help of this revised modified surgical procedure, the syndesmosis complex was successfully preserved after the tumor resection was achieved. The observation of the clinical outcome in this study proved that this new surgical technique is able to significantly improve the stability of the ankle joint and the long-term function. To our knowledge, this is the first time to perform the excision of the distal tibial interosseous osteochondroma involving the fibula without interrupting the inferior syndesmotic complex especially the AITFL. We believe that this strategy may pave a new way for the further clinical application for these patients with distal tibiofibular interosseous osteochondroma to obtain a more stable ankle joint after surgery.

## Figures and Tables

**Table 1:** The clinical information of the patients.

Case	Gender	Age	Side	Radiographic test	Symptoms
1	F	17	L	X-ray, CT	Pain
2	M	19	L	X-ray, CT	Pain
3	F	29	L	X-ray, CT	Pain when wearing the boots
4	F	21	R	MRI, CT	Painful lump
5	M	18	R	X-ray, CT	Pain
6	M	32	L	X-ray, CT	Restriction of joint motion
7	F	20	R	X-ray, CT	Painful lump
8	M	43	L	X-ray, CT	Restriction of joint motion
9	M	28	R	X-ray, CT	Painful lump, restriction of joint motion
10	M	31	R	X-ray, CT	Painful lump, restriction of joint motion
11	M	24	L	X-ray, CT	Painful lump

M: male; F: female; L: left; R: right; CT: computerized tomographic scanning; MRI: magnetic resonance imaging.

**Table 2:** Visual Analogue Scale score and grade.

Case	Period					
	Preoperative		3 months post operation		1 year post operation	
	Score	Grade	Score	Grade	Score	Grade
1	9	Poor	4	Good	1	Good
2	8	Poor	7	Poor	4	Fair
3	7	Poor	3	Good	1	Good
4	7	Poor	2	Good	0	Good
5	7	Poor	2	Good	1	Good
6	9	Poor	4	Good	2	Good
7	7	Poor	3	Good	3	Good
8	6	Fair	5	Fair	2	Good
9	8	Poor	5	Fair	1	Good
10	8	Poor	4	Fair	2	Good
11	6	Fair	2	Good	1	Good
Average score	7.45 ± 2.15		3.73 ± 0.33^∗^		1.73 ± 0.27^#^	
Good rate	—		63.6%		90.9%^∗^	

Compared with preoperative VAS score, ^∗^*P* < 0.05, ^#^*P* < 0.01. Compared with 3 months follow-up, ^∗^*P* < 0.05.

**Table 3:** AOFAS score and grade.

Case	Period					
	Before operation		3 months post operation		1 year post operation	
	Score	Grade	Score	Grade	Score	Grade
1	49	Poor	71	Fair	95	Excellent
2	22	Poor	48	Poor	89	Good
3	42	Poor	84	Good	96	Excellent
4	37	Poor	73	Fair	94	Excellent
5	57	Fair	89	Good	97	Excellent
6	34	Poor	67	Fair	91	Excellent
7	42	Poor	72	Fair	92	Excellent
8	61	Fair	86	Good	95	Excellent
9	63	Fair	82	Good	91	Excellent
10	58	Fair	79	Good	94	Excellent
11	45	Poor	88	Good	96	Excellent
Average score	47.27 ± 5.27		80.99 ± 4.33^∗^		93.63 ± 6.91^#^	

Compared with preoperative VAS score, ^∗^*P* < 0.05, ^#^*P* < 0.01.

## Data Availability

1. The [Functional outcome of the patients enrolled in our study] data used to support the findings of this study data are included within the article. 2. The [raw data of all the functional assessment of the patients enrolled in our study] data used to support the findings of this study are available from the corresponding author upon request.

## References

[1] Unni K. K. (2001). Cartilaginous lesions of bone. *Journal of Orthopaedic Science*.

[2] Chin K. R., Kharrazi F. D., Miller B. S., Mankin H. J., Gebhardt M. C. (2000). Osteochondromas of the distal aspect of the tibia or fibula. Natural history and treatment. *The Journal of Bone and Joint Surgery American Volume*.

[3] Kitsoulis P., Galani V., Stefanaki K. (2008). Osteochondromas: review of the clinical, radiological and pathological features. *In Vivo*.

[4] Ismail B. E., Kissel C. G., Husain Z. S., Entwistle T. (2008). Osteochondroma of the distal tibia in an adolescent: a case report. *The Journal of Foot and Ankle Surgery*.

[5] Sorensen B. W., Mikkelsen P. (2012). Arthrodesis of the distal tibiofibular joint for an osteochondroma in the fibula encroaching on the distal tibia and involving the talocrural joint: a case report. *The Journal of Foot and Ankle Surgery*.

[6] Hermans J. J., Beumer A., de Jong T. A., Kleinrensink G. J. (2010). Anatomy of the distal tibiofibular syndesmosis in adults: a pictorial essay with a multimodality approach. *Journal of Anatomy*.

[7] Dalmau-Pastor M., Malagelada F., Kerkhoffs G. M. M. J., Karlsson J., Manzanares M. C., Vega J. (2020). The anterior tibiofibular ligament has a constant distal fascicle that contacts the anterolateral part of the talus. *Knee Surgery, Sports Traumatology, Arthroscopy: Official Journal of the ESSKA*.

[8] Vila-Rico J., Sanchez-Morata E., Vacas-Sanchez E., Ojeda-Thies C. (2018). Anatomical arthroscopic graft reconstruction of the anterior tibiofibular ligament for chronic disruption of the distal syndesmosis. *Arthroscopy Techniques*.

[9] Kitaoka H. B., Alexander I. J., Adelaar R. S. (2017). Clinical rating systems for the ankle-hindfoot, midfoot, hallux, and lesser toes. *Foot & Ankle International*.

[10] Heller G. Z., Manuguerra M., Chow R. (2016). How to analyze the visual analogue scale: myths, truths and clinical relevance. *Scandinavian Journal of Pain*.

[11] Thakur G. B., Jain M., Bihari A. J., Sriramka B. (2012). Transfibular excision of distal tibial interosseous osteochondroma with reconstruction of fibula using Sofield's technique - a case report. *Journal of Clinical Orthopaedics and Trauma*.

[12] Herrera-Perez M., Aciego de Mendoza M., de Bergua-Domingo J. M., Pais-Brito J. L. (2013). Osteochondromas around the ankle: report of a case and literature review. *International Journal of Surgery Case Reports*.

